# Additive and Dominance Genome-Wide Association Studies Reveal the Genetic Basis of Heterosis Related to Growth Traits of Duhua Hybrid Pigs

**DOI:** 10.3390/ani14131944

**Published:** 2024-06-30

**Authors:** Jiakun Qiao, Kebiao Li, Na Miao, Fangjun Xu, Pingping Han, Xiangyu Dai, Omnia Fathy Abdelkarim, Mengjin Zhu, Yunxiang Zhao

**Affiliations:** 1 Key Lab of Agricultural Animal Genetics, Breeding, and Reproduction of Ministry of Education, Huazhong Agricultural University, Wuhan 430070, China; jkqiao@webmail.hzau.edu.cn (J.Q.); miaona@webmail.hzau.edu.cn (N.M.); fjxu@webmail.hzau.edu.cn (F.X.); hping@webmail.hzau.edu.cn (P.H.); daixy@webmail.hzau.edu.cn (X.D.); omniafathyabdelkarim@gmail.com (O.F.A.); zhumengjin@mail.hzau.edu.cn (M.Z.); 2 School of Life Science and Engineering, Foshan University, Foshan 528000, China; bb973900622@163.com; 3 The Cooperative Innovation Center for Sustainable Pig Production, Huazhong Agricultural University, Wuhan 430070, China; 4 College of Animal Science and Technology, Guangxi University, Nanning 530004, China

**Keywords:** Duhua hybrid pigs, heterosis, additive effect, dominance effect, partial genetic value, genome-wide association studies

## Abstract

**Simple Summary:**

Heterosis has been extensively utilized in the genetic breeding and production of pigs, necessitating further analysis of its potential genetic mechanisms. The objective of this study was to elucidate the genetic architecture of heterosis by employing a hybrid model incorporating commercial and native pigs. In this study, we observed that the growth traits of the Duhua hybrid pigs exhibited significantly superior performance compared with their parents’ average performance. Furthermore, we revealed the influence of additive and dominance genetic effects on the growth traits of Duhua hybrid pigs. Our findings provide an insight into the genetic basis of the heterosis of Duhua pigs and offer a reference for the utilization of local pig breeds.

**Abstract:**

Heterosis has been extensively used for pig genetic breeding and production, but the genetic basis of heterosis remains largely elusive. Crossbreeding between commercial and native breeds provides a good model to parse the genetic basis of heterosis. This study uses Duhua hybrid pigs, a crossbreed of Duroc and Liangguang small spotted pigs, as materials to explore the genetic basis underlying heterosis related to growth traits at the genomic level. The mid-parent heterosis (MPH) analysis showed heterosis of this Duhua offspring on growth traits. In this study, we examined the impact of additive and dominance effects on 100 AGE (age adjusted to 100 kg) and 100 BF (backfat thickness adjusted to 100 kg) of Duhua hybrid pigs. Meanwhile, we successfully identified SNPs associated with growth traits through both additive and dominance GWASs (genome-wide association studies). These findings will facilitate the subsequent in-depth studies of heterosis in the growth traits of Duhua pigs.

## 1. Introduction

Heterosis refers to the superiority of offspring from the crossbreeding of two parents with distinct genetic backgrounds in growth, stress resistance, and adaptability to both their parental lines [1,2]. Heterosis has found extensive applications in pig breeding, particularly for improving growth traits such as age at a specific weight (AGE) and backfat thickness (BF), and it can effectively improve growth rate, meat quality, and disease resistance [3,4,5]. These traits are pivotal economic traits, and they are frequently employed as economic indicators for evaluating productivity [6,7,8]. Therefore, it is imperative to analyze the genetic background underlying heterosis in porcine growth traits.

The genetic architectures of animal growth traits are complex and often involve multiple genes with additive and non-additive effects, and the non-additive effects play an important role in forming heterosis [9,10]. With the advancement of genotyping technology, genome-wide association studies (GWASs) have been widely used for analyzing complex growth traits [11,12,13]. GWASs are a method used to identify the genetic locuses associated with traits by assessing the significance of the association between genome-wide genetic markers and phenotypic variation [14,15]. Most existing GWAS analyses predominantly focus on additive effects, and non-additive effects tend to be overlooked. However, identifying the non-additive effects associated with complex traits is crucial for comprehending the genetic architectures of complex traits [16]. In recent years, many studies have incorporated non-additive and additive effects in genome prediction [17,18,19] and GWAS analysis [20,21] to explore genetic effects, thereby deciphering genetic mechanisms. An additive GWAS can provide a robust analytical framework for investigating the complex traits of a hybrid population, while a non-additive GWAS can further elucidate the underlying genetic mechanism.

Non-additive genetic effects are the key to the formation of heterosis [22]. In pig breeding, numerous studies have investigated the impacts of different genetic effects on growth traits through splitting genetic effects [17,18,19,20,21]. With the development of production technology, native pig breeds have been widely utilized in hybrid production due to their exceptional and distinctive quality [23,24]. Crossbreeding between commercial and native pigs offers valuable models for dissecting the genetic basis of heterosis. The investigation of the hybrids between commercial and native pigs is instrumental in comprehensively analyzing the genetic background of heterosis in pig breeding and furnishing an empirical foundation for harnessing and exploiting the distinctive attributes of native pigs.

The hybridized combination of Duroc and Liangguang small spotted pigs has been applied to the economic market. Duroc is a commonly utilized terminal sire line due to its advantageous traits such as rapid growth and high lean meat content [25]. The Liangguang small spotted pig, renowned for its excellent meat quality and high reproductive performance, plays an important role in meeting market demand and represents a prominent native breed in the Guangxi and Guangdong regions of China [26]. However, the utilization and analysis of heterosis in Duhua hybrids still require numerous studies to provide sufficient empirical support.

The objective of this study was to investigate the genetic basis of heterosis in the growth traits of Duhua pigs, a crossbreed between Duroc and Liangguang small spotted pigs. We comprehensively analyzed the genetic effects on two growth traits of the Duhua pigs, namely, 100 AGE (age adjusted to 100 kg) and 100 BF (backfat thickness adjusted to 100 kg), and found the presence of significant dominance effects. Further, we successfully identified additive and dominance SNPs associated with growth traits throughout and mapped genes involved in muscle and fat metabolism. Our findings, presented herein, provide compelling evidence for the presence of heterosis in Duhua hybrid pigs, thereby enhancing our understanding of heterosis in pig breeding and offering valuable insights for the utilization of native pig hybridization.

## 2. Materials and Methods

All the data were obtained from routine breeding activities.

### 2.1. Animals

We selected Duroc boars with high lean meat rates and fast growth rates to cross with Liangguang small spotted pigs with good fattening performance and high reproductive performance, and all pigs were in good health. The crossbreeding experiment involved the utilization of 6 Duroc boars and 24 Liangguang small spotted sows, resulting in the production of 171 Duhua hybrid pigs (86 males and 85 females). All the animals were derived from Guangdong YIHAO Food Co., Ltd. (Guangzhou, Guangdong, China). All the Duhua hybrid pigs were raised under identical conditions.

### 2.2. Phenotype Data

The initial weights, BFs, and AGEs at the beginning of experiment and final weights, BFs, and AGEs at the end of the experiment for the Duhua pigs were measured and recorded. Then, the AGEs and BFs at the end of the experiment in this study were adjusted to 100 AGE (age adjusted to 100 kg) and 100 BF (backfat thickness adjusted to 100 kg), respectively, according to the following formulae [16]:

(i) 100 AGE (age adjusted to 100 kg):

(1) $$100\;AGE=AGE+\left(100-wt\right)\times \left(\frac{AGE-A}{wt}\right)$$

where AGE represents the age at the end of the experiment; wt represents the weight at the end of the experiment; and A is the correction coefficient for sires and sows ($A_{sire}$ = 50.775 and $A_{sow}$ = 46.415).

(ii) 100 BF (BF adjusted to 100 kg):

(2) $$100\;BF=BF+\left(100-wt\right)\times \left(\frac{BF}{wt-B}\right)$$

where BF indicates the backfat thickness at the end of the experiment; wt denotes the weight at the end of the experiment; and B is the correction coefficient for sires and sows ($B_{sire}$ = −7.277 and $B_{sow}$ = −9.440).

### 2.3. Processing of Genotype Data

Ear samples of the Duhua hybrid pigs were collected for DNA extraction. Subsequently, the extracted DNA was genotyped using a Porcine 80 K Functional Variants Genotyping Array (Wuhan Yingzi Gene Technology Co., Ltd., Wuhan, China), and a total of 204,489 SNPs (single-nucleotide polymorphisms) were obtained. Subsequently, we excluded SNPs located on the X chromosome and conducted quality control on the remaining SNPs using PLINK1.9 software [27]. The SNPs with call rates of <0.95 were filtered. Then, the missing genotype data were imputed by Beagle5.4 software, with a haplotype reference panel unused [28]. The SNPs with minor allele frequencies (MAFs) of <0.01 were removed after data imputation. After genotype data processing, 112,643 SNPs were retained for subsequent analysis.

### 2.4. Calculation of Mid-Parent Heterosis

Mid-parent heterosis (MPH) [29], an index of heterosis, was calculated according to the following formula:

(3) $$MPH=\frac{F1-(P1+P2)/2}{(P1+P2)/2}\times 100\%$$

Here, MPH is the mid-parent heterosis value; F1 is the phenotypic observation values of the hybrid offspring Duhua pigs; and P1 and P2 are the phenotypic observation values of the same traits of both parents, respectively.

### 2.5. Estimation of Genetic Components

The genetic variance components were estimated using the semi-parametric methods (Bayesian-reproducing kernel Hilbert space regressions, RKHSs) in the BGLR package [30,31]. The model can be written as

(4) $$y=Xb+Z_{a}u_{a}+Z_{d}u_{d}+e$$

where y is the vector of the phenotypes (100 AGE and 100 BF); b is the vector of the fixed effect; $u_{a}$ and $u_{d}$ are the vectors of the additive and dominance genetic effects; X, $Z_{a}$, and $Z_{d}$ are the design matrices for the fixed effects, additive genetic effects, and dominance genetic effects, respectively; and e is the vector of the residual error. In the model, additive, dominance, and residual effects were assumed to be $u_{a}\;\sim \;N\left(0\;,\;G_{a}\sigma_{a}^{2}\right)$, $u_{d}\;\sim \;N\left(0\;,\;G_{d}\sigma_{d}^{2}\right)$, and $e\;\sim \;N\left(0\;,\;I\sigma_{e}^{2}\right)$, respectively. $\sigma_{a}^{2}$, $\sigma_{d}^{2}$, and $\sigma_{e}^{2}$ are additive genetic variance, dominance genetic variance, and residual variance, and *I* is an identity matrix. $G_{a}$ is the additive genetic relationship matrix, which was constructed using the formula $G_{a}=\frac{ZZ^{\prime }}{\sum_{1}^{m}2p_{i}{(1-p}_{i})}$ [32,33], where *Z* is the standardized matrix of the additive genotypes and genotypes AA, Aa, and aa are encoded by 0, 1, and 2, respectively. $G_{d}$ is the dominance genetic relationship matrix, which was constructed using the formula $G_{d}=\frac{{WW}^{\prime }}{\sum_{1}^{m}{\left[2p_{i}{(1-p}_{i})\right]}^{2}}$ [19], where *W* is the standardized matrix of the dominance genotypes and genotypes AA, Aa, and aa are encoded by 0, 1 and 0, respectively.

### 2.6. Estimation of Partial Genetic Values

In this study, we partitioned the genetic effects into additive and dominance effects and estimated partial genetic values (PGVs, including additive and dominance PGVs) based on partial genetic effects. Subsequently, these PGVs were employed as novel phenotypes for further analysis. The PGVs were estimated according to the following formulae:

(5) $$y_{a}=Z_{a}u_{a}$$

(6) $$y_{d}=Z_{d}u_{d}$$

where $y_{a}$ is a vector of the additive PGVs; $y_{d}$ is a vector of the dominance PGVs; and $Z_{a}$, $u_{a}$, $Z_{d}$, and $u_{d}$ are the same as described in Formula (4).

### 2.7. Additive and Dominance Genome-Wide Association Studies

The E-GWAS [34] strategy was implemented to integrate five statistical models, namely MLM [35], REMMAX [36], MLMM [37], FarmCPU [38,39], and BLINK [40], for conducting both additive and dominance GWAS analyses.

Then, the Bonferroni correction [41] was implemented to define the significant threshold. To avoid missing the true hints of linkage, the genome-wide significant and suggestive thresholds were defined as p=0.05/N and p=1/N, respectively, where N is the number of analyzed SNPs.

### 2.8. Identification and Functional Analysis of Candidate Genes

The gene annotation information from the *Sus scrofa* genome (version v11.1) in the Ensembl database [42] was utilized to identify genes. We further performed gene ontology (GO) enrichment analysis and Kyoto Encyclopedia of Genes and Genomes (KEGG) analysis on the KOBAS 3.0 website “http://kobas.cbi.pku.edu.cn (accessed on 10 March 2024)” to elucidate the functions of these candidate genes [43,44]. We also explored their functions through the PubMed website “https://pubmed.ncbi.nlm.nih.gov/ (accessed on 10 March 2024)”.

## 3. Results

### 3.1. Mid-Parent Heterosis in Two Traits, 100 AGE and 100 BF, of Duhua Pigs

**Figure 1:**
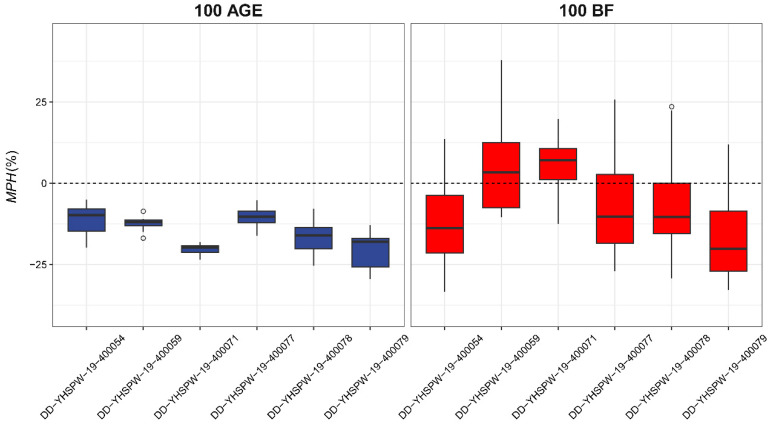
Mid-parent heterosis (MPH) in two traits, 100 AGE and 100 BF, of Duhua hybrid pigs. The Duhua hybrid pigs were divided into six groups by paternity, and the boxes represent the distribution of individual heterosis in different groups. The dotted line indicates that the MPH is 0.

The Duhua pigs were divided into six groups based on paternity. Figure 1 shows the mid-parent heterosis of these six hybrid groups in terms of the traits 100 AGE and 100 BF. As for 100 AGE, all six groups exhibited heterosis at both the population and individual levels. As for 100 BF, the offspring outperformed the mean performances of both parents at the population level in four groups.

### 3.2. Estimation of Genetic Components and Heritability

We partitioned the genetic effects of the Duhua pigs into additive and dominance effects and employed the RKHS method of the BGLR package to estimate the genetic components of 100 AGE and 100 BF, respectively (Table 1). The results revealed that the dominance variance components accounted for 51.73 and 48.14% of the total genetic variance in the two traits, respectively, confirming the presence of heterosis in the Duhua hybrid pigs.

### 3.3. Genome-Wide Association Studies

We conducted genome-wide association study (including both additive and dominance GWAS) analyses of three traits using the E-GWAS strategy. In this study, we proposed utilizing additive and dominance effects separately to estimate partial genetic values (PGVs) and used the PGVs as novel phenotypes for GWAS analysis to identify more significant SNPs.

### 3.4. E-GWAS on Additive and Dominance Simulations

We first performed additive and dominance simulations on the population to assess the applicability of the E-GWAS. In the process, the GAPIT3 [45] package was utilized to conduct one hundred additive and one hundred dominance simulated phenotypes, each with a heritability of 0.5. In each simulation, 20 SNPs were randomly selected as quantitative trait nucleotides (QTNs) from genotypes with additive or dominance effects. The effect of each QTN was derived from a standard normal distribution.

**Figure 2:**
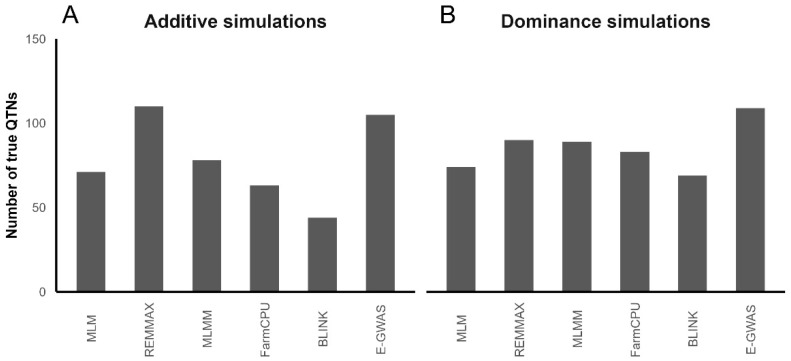
Results of different models in additive and dominance simulations. Each bar represents the number of true QTNs identified by various models.

In additive simulations, both the REMMAX model and the E-GWAS demonstrated significantly superior performance compared to the other four models (Figure 2A). The dominance simulations demonstrated that the E-GWAS outperformed the five single models in terms of identifying true QTNs (Figure 2B). The simulation results demonstrated that the E-GWAS method was well-suited for analyzing this dataset, and it exhibited superior stability compared to the single model.

### 3.5. Analysis of Additive Effects

**Figure 3:**
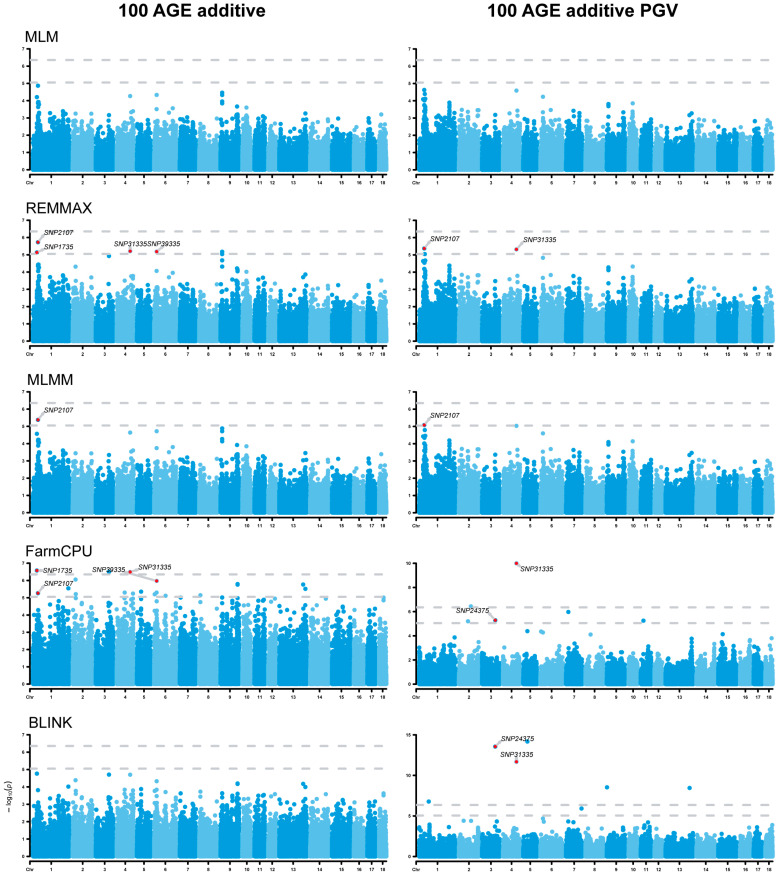
Manhattan plots of additive genome-wide association studies (GWASs) for 100 AGE and 100 AGE additive PGVs. The rows represent the GWAS results of different models, and the annotated SNPs are the significant ones identified by the E-GWAS. The dotted lines represent the Bonferroni correction thresholds.

The additive GWAS revealed four additive SNPs linked to the 100 AGE trait, positioned on chromosomes ssc1, ssc2, and ssc3 (Figure 3). Specifically, two SNPs on scc1 are in the *SLC2A12* (solute carrier family 2 member 12) gene and the *RNF217* (ring finger protein 217) gene, respectively. Additionally, the *WARS2* (tryptophanyl tRNA synthetase 2, mitochondrial) gene and the *GINS3* (GINS complex subunit 3) gene were found in the close vicinity of two SNPs positioned on ssc4 and ssc6 (Table 2). Utilizing additive PGVs as a novel phenotype, three additive SNPs were identified to be associated with 100 AGE (Figure 3). Two of these SNPs, positioned on ssc1 and ssc2, overlapped with the original phenotype results, while one new SNP, positioned on ssc3, was discovered through the 100 AGE additive PGV analysis. Additionally, the *PKDCC* (protein kinase domain containing, cytoplasmic) gene was found near the locus located on ssc3 (Table 2).

**Figure 4:**
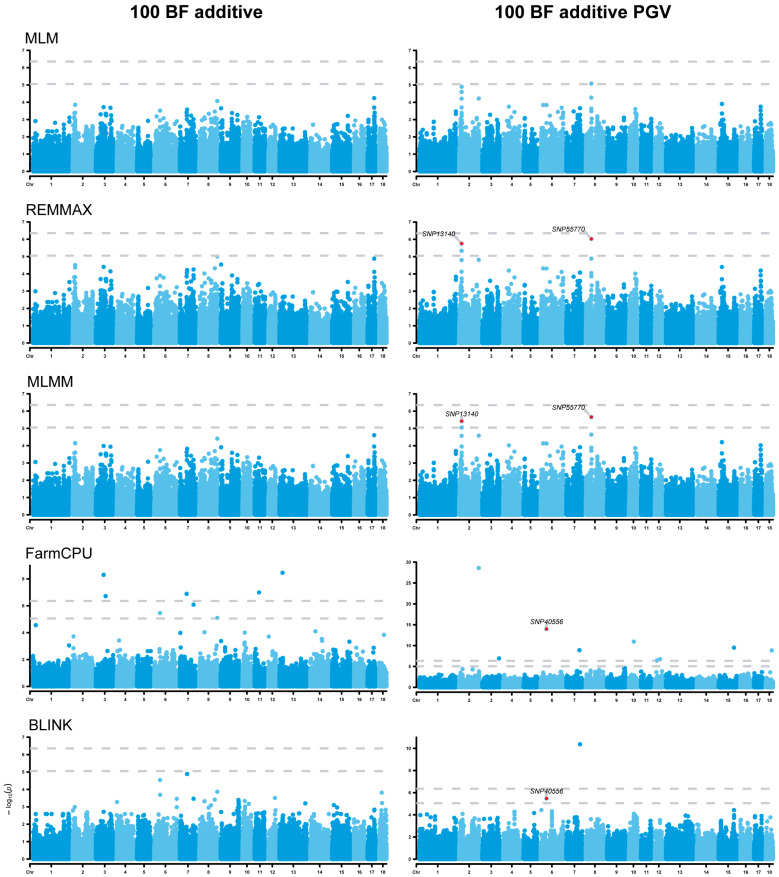
Manhattan plots of additive genome-wide association studies (GWASs) for 100 BF and 100 BF additive PGVs. The rows represent the GWAS results of different models, and the annotated SNPs are the significant ones identified by the E-GWAS. The dotted lines represent the Bonferroni correction thresholds.

For the 100 BF additive GWAS analysis, no significant SNPs associated with the original phenotype were detected by the E-GWAS, whereas three significant SNPs were identified in 100 BF additive PGVs on ssc2, ssc6, and ssc8 (Figure 4). These SNPs are located proximally to the *API5* (apoptosis inhibitor 5), *FAM187B* (family with sequence similarity 187 member B), and *MAP9* (microtubule associated protein 9) genes (Table 2).

### 3.6. Analysis of Dominance Effects

**Figure 5:**
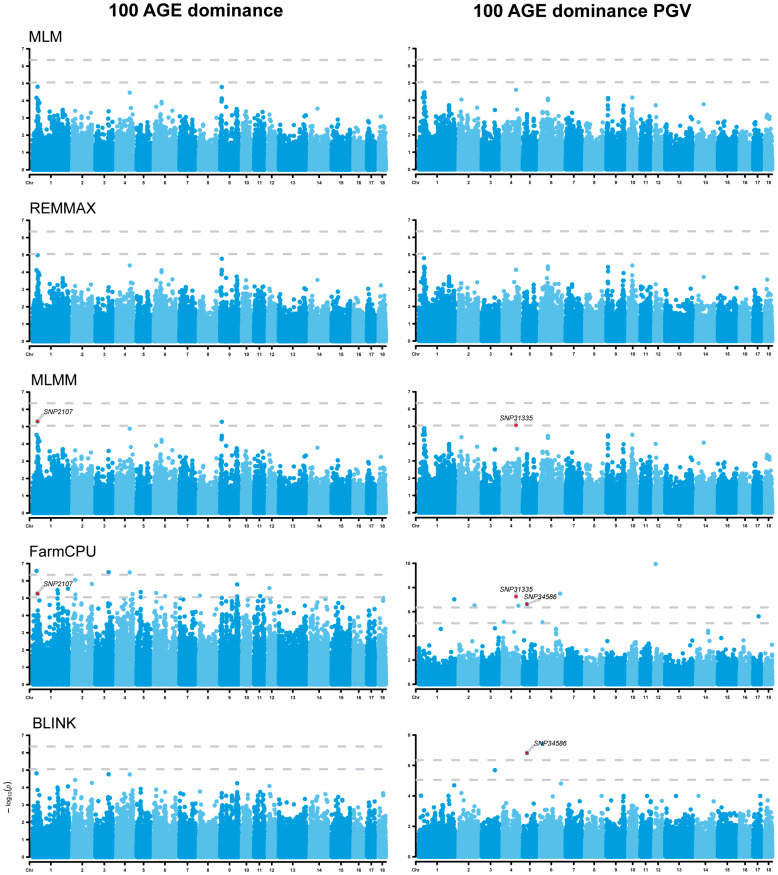
Manhattan plots of dominance genome-wide association studies (GWASs) for 100 AGE and 100 AGE dominance PGVs. The rows represent the GWAS results of different models, and the annotated SNPs are the significant ones identified by the E-GWAS. The dotted lines represent the Bonferroni correction thresholds.

**Figure 6:**
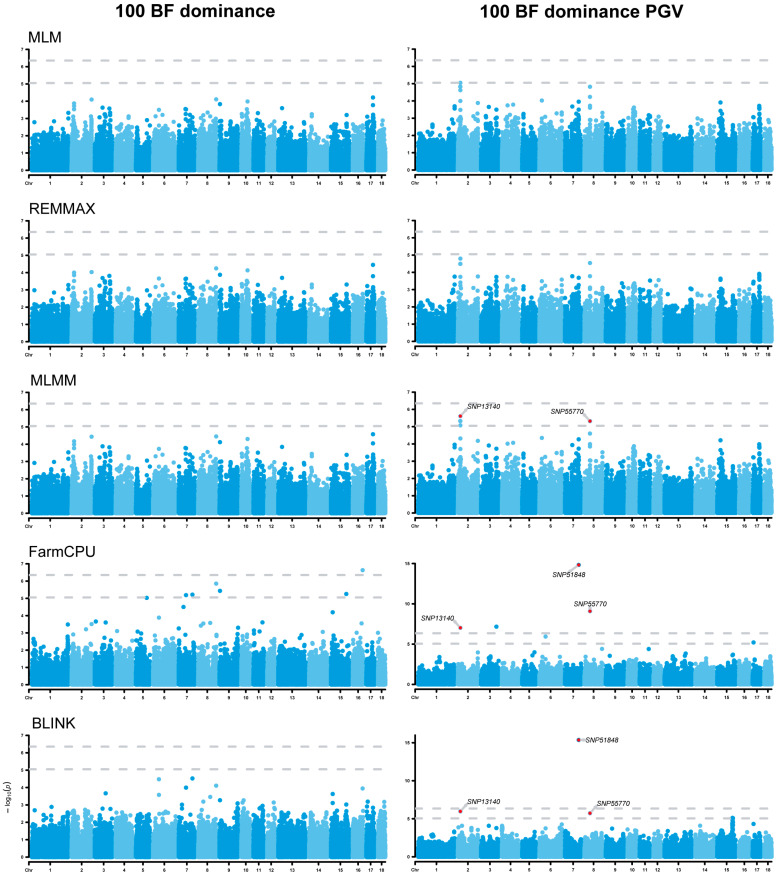
Manhattan plots of dominance genome-wide association studies (GWASs) for 100 BF and 100 BF dominance PGVs. The rows represent the GWAS results of different models, and the annotated SNPs are the significant ones identified by the E-GWAS. The dotted lines represent the Bonferroni correction thresholds.

The dominance GWAS results showed that one dominance SNP (positioned on chromosome ssc1) was identified to be associated with the 100 AGE trait (Figure 5). The GWAS results of the dominance PGVs showed that a total of two dominance SNPs on ssc4 and ssc5 were identified to be associated with the 100 AGE dominance PGV (Figure 5), while three SNPs were found to be associated with the 100 BF dominance PGV on ssc2, ssc7, and ssc8 (Figure 6). Notably, one novel SNP was discovered for each of the two traits in comparison to the additive GWAS. The six genes, namely *RNF217*, *WARS2*, *GRIP1* (glutamate receptor interacting protein 1), *API5*, *NRXN3* (neurexin 3), and *MAP9*, located in/near these SNPs, were further identified as potential candidates associated with these traits (Table 3).

### 3.7. Functional Enrichment of Candidate Genes

GO enrichment analysis, KEGG analysis, and extensive literature on PubMed showed that these genes were involved in the limb morphogenesis, glucocorticoid receptor binding, embryonic digestive tract development, glucose transmembrane transport, mitotic cytokinesis, skeletal system development, and multicellular organism growth processes (Table S1). These processes are closely related to the growth and development of the body.

## 4. Discussion

This study explored the genetic basis of the growth trait heterosis of Duhua pigs based on whole-genome information. The growth traits of the Duhua pigs were superior to those of the parent sows (Table S2) in six hybrid groups with different sires and exceeded the average levels of the parent groups. Since heterosis is governed by non-additive genetic components, we examined the genomic genetic structure associated with the growth traits of the Duhua pigs. The analysis of the genetic variance in the 100 AGE and 100 BF traits unveiled significant dominance effects in hybrids.

GWASs have been widely used for phenotype–genotype association analysis. Numerous GWAS models have been developed based on different statistical assumptions. However, given the diversity in the genetic architectures of complex traits, no single model can be considered optimal for all traits. Previous studies have demonstrated that combining different GWAS models can enhance the detection rates and statistical robustness of major quantitative trait locuses (QTLs) [46,47,48]. Considering the limited sample size, we utilized the E-GWAS [34] strategy to integrate multiple GWAS models for phenotype–genotype association analysis. Additionally, our additive and dominance simulations on this dataset indicated that the E-GWAS is more stable than a single model in small samples.

To avoid overlooking growth trait-related SNPs, we also proposed introducing PGVs (partial genetic values), which were constructed based on the genetic effect values of specific pleiotropic genes to the GWAS as a novel phenotype to identify growth trait-associated SNPs. Estimated breeding value (EBV) has been extensively employed as a response variable in GWASs [49,50,51,52]. The genetic effects were partitioned in this study, and the PGVs were estimated as the response variables based on partial genetic effects. The results showed that the analysis based on PGVs could accurately identify certain SNPs that were consistent with the original phenotype while also detecting previously unidentified SNPs. The findings suggested that incorporating PGVs as a novel phenotype can enhance GWAS analysis and facilitate the identification of trait-associated SNPs, thereby enabling a more comprehensive understanding of the genetic mechanisms underlying traits.

Using additive and dominance genome-wide association studies (GWASs), we have successfully identified eight additive SNPs (five SNPs for 100 AGE and three for 100 BF), as well as six dominance SNPs (three SNPs for 100 AGE and three for 100 BF), that are associated with growth traits in hybrid populations. Subsequently, we screened the genes near these significant SNPs. Four of these genes, *SLC2A12*, *GINS3*, *PKDCC*, and *FAM187B*, were exclusively identified through additive analysis. *SLC2A12* belongs to a family of transporters that catalyze the uptake of sugars through facilitated diffusion [53,54]. Previous studies have demonstrated the significant impact of the *SLC2A12* gene on loin eye area and fatness traits in Berkshire and/or Yorkshire pigs [55]. The *GINS3* gene functions as a component of DNA helicase complexes [56] and exhibits downregulation in metabolically unhealthy obese adults [57]. The *PKDCC* gene has been reported to exhibit a significant association with bone development in both humans and mice [58,59]. The application of dominance analysis revealed that the genes *GRIP1* and *NRXN3* emerged as promising candidates for growth traits in Duhua hybrid pigs. *GRIP1* has been identified as a regulator of the pig feed conversion rate [60]. The *NRXN3* gene has been found to be associated with general obesity [61]. The *RNF217*, *WARS2*, *API5*, and *MAP9* genes were identified in both additive and dominance analyses. In these genes, RNF217 regulates iron homeostasis by degrading the iron exporter ferroportin [62]. The *WARS2* gene is related to obesity [63]. The *API5* gene has been reported as a suitable reference gene for gene expression analysis in porcine skeletal muscle development [64].

Although this study analyzed the genetic background of Duhua hybrid pigs and successfully identified significant SNPs through additive and dominant effect analysis, further analysis is warranted. In future studies, the comprehensive analysis of Duhua hybrid pigs’ heterosis can be enhanced by expanding the population data and considering non-additive effects beyond dominance effects. The comprehensive use of multi-omics data is helpful for us to analyze this hybrid population from multiple dimensions.

## 5. Conclusions

In this study, we analyzed the genetic background of growth trait heterosis in Duhua hybrids of Duroc and Liangguang small spotted pigs at the genome level. The observation of significant dominance effects in the hybrid offspring provides compelling evidence for the presence of heterosis. We further identified several genes involved in muscle and fat metabolism through GWAS analysis. In conclusion, our findings elucidate the genetic background of heterosis in Duhua hybrid pigs, providing an empirical foundation for pig crossbreeding and facilitating a comprehensive comprehension of the application and significance of heterosis in pig breeding. In addition, this proposal also includes the construction of partial genetic values (PGVs) for extended analysis, thereby enriching the genomic analysis of complex traits in hybrid populations.

## Figures and Tables

**Table 1:** Estimation of variance components and heritability for 100 AGE and 100 BF.

Trait	$\sigma_{a}^{2}$ (SE)	$\sigma_{d}^{2}$ (SE)	$\sigma_{e}^{2}$ (SE)	$h_{a}^{2}$ (SE)	$h_{d}^{2}$ (SE)	$\frac{\sigma_{d}^{2}}{\sigma_{a}^{2}+\sigma_{d}^{2}}$
100 AGE	5.3080 (0.2004)	5.6899 (0.2718)	8.1597 (0.1523)	0.3322 (0.0125)	0.3561 (0.017)	0.5173
100 BF	3.8868 (0.1189)	3.6087 (0.1518)	6.4086 (0.1007)	0.2523 (0.0077)	0.2342 (0.0098)	0.4814

Notes: $\sigma_{a}^{2}$, additive genetic variance; $\sigma_{d}^{2}$, dominance genetic variance; $\sigma_{e}^{2}$, residual variance; $h_{a}^{2}$, additive (narrow-sense) heritability; $h_{d}^{2}$, dominance heritability; SE, standard error; $\frac{\sigma_{d}^{2}}{\sigma_{a}^{2}+\sigma_{d}^{2}}$, ratio of dominance genetic variance to total genetic variance.

**Table 2:** Significant additive SNPs associated with growth traits.

Trait	SNP	Chr	Pos (bp)	Model	Gene	Distance (bp)
100 AGE	SNP1735	1	29,864,163	REMMAX, FarmCPU	*SLC2A12*	0
	SNP2107	1	37,901,112	REMMAX, MLMM, FarmCPU	*RNF217*	0
	SNP31335	4	102,102,250	REMMAX, FarmCPU	*WARS2*	−219,180
	SNP39335	6	20,128,919	REMMAX, FarmCPU	*GINS3*	+33,717
100 AGE additive PGV	SNP2107	1	37,901,112	REMMAX, MLMM	*RNF217*	0
	SNP24375	3	98,862,214	FarmCPU, BLINK	*PKDCC*	+593,661
	SNP31335	4	102,102,250	REMMAX, FarmCPU, BLINK	*WARS2*	−219,180
100 BF additive PGV	SNP13140	2	19,545,736	REMMAX, MLMM	*API5*	−551,496
	SNP40556	6	44,729,418	FarmCPU, BLINK	*FAM187B*	+12,795
	SNP55770	8	42,734,254	MLM, REMMAX, MLMM	*MAP9*	+390

Notes: Chr, chromosome; Pos, SNP position; Model, GWAS models with the identified significant SNP; Distance, distances between gene and SNP; + indicates that the gene is upstream of the SNP, − indicates that the gene is downstream of the SNP, and 0 indicates that the SNP is in the gene.

**Table 3:** Significant dominance SNP(s) associated with growth traits.

Trait	SNP	Chr	Pos (bp)	Model	Gene	Distance (bp)
100 AGE	SNP2107	1	37,901,112	MLMM, FarmCPU	*RNF217*	0
100 AGE dominance PGV	SNP31335	4	102,102,250	MLMM, FarmCPU	*WARS2*	−219,180
	SNP34586	5	30,709,370	FarmCPU, BLINK	*GRIP1*	0
100 BF dominance PGV	SNP13140	2	19,545,736	MLMM, FarmCPU, BLINK	*API5*	−551,496
	SNP51848	7	102,484,784	FarmCPU, BLINK	*NRXN3*	−546,486
	SNP55770	8	42,734,254	MLMM, FarmCPU, BLINK	*MAP9*	+390

Notes: Chr, chromosome; Pos, SNP position; Model, GWAS models with the identified significant SNP; Distance, distances between gene and SNP; + indicates that the gene is upstream of the SNP, − indicates that the gene is downstream of the SNP, and 0 indicates that the SNP is in the gene.

## Data Availability

The genotype data analyzed in this study are accessible on figshare (https://doi.org/10.6084/m9.figshare.24899142). The phenotype data are not publicly accessible due to their sourcing from Guangdong YIHAO Food Co., Ltd., but they can be obtained from the corresponding author upon reasonable request.

## Supplementary Materials

The following supporting information can be downloaded at: https://www.mdpi.com/article/10.3390/ani14131944/s1, Table S1: Functional annotation of candidate genes; Table S2: Summary statistics of phenotypic means for Duhua hybrid pigs and small spotted pigs of six groups.

## References

[1] Shull G.H. (1908). The Composition of a Field of Maize. J. Hered..

[2] Shull G.H. (1948). What Is Heterosis. Genetics.

[3] Mohammadpanah M., Mehrgardi A.A., Gilbert H., Larzul C., Mercat M.J., Esmailizadeh A., Momen M., Tusell L. (2022). Genic and non-genic SNP contributions to additive and dominance genetic effects in purebred and crossbred pig traits. Sci. Rep..

[4] Esfandyari H., Thekkoot D., Kemp R., Plastow G., Dekkers J. (2020). Genetic parameters and purebred-crossbred genetic correlations for growth, meat quality, and carcass traits in pigs. J. Anim. Sci..

[5] Steyn Y., Lourenco D.A., Chen C.Y., Valente B.D., Holl J., Herring W.O., Misztal I. (2021). Optimal definition of contemporary groups for crossbred pigs in a joint purebred and crossbred genetic evaluation. J. Anim. Sci..

[6] Fontanesi L., Schiavo G., Galimberti G., Calò D.G., Russo V. (2014). A genomewide association study for average daily gain in Italian Large White pigs. J. Anim. Sci..

[7] Ding R.R., Yang M., Wang X.W., Quan J.P., Zhuang Z.W., Zhou S.P., Li S.Y., Xu Z., Zheng E.Q., Cai G.Y. (2018). Genetic Architecture of Feeding Behavior and Feed Efficiency in a Duroc Pig Population. Front. Genet..

[8] Tang Z.S., Xu J.Y., Yin L.L., Yin D., Zhu M., Yu M., Li X.Y., Zhao S.H., Liu X.L. (2019). Genome-Wide Association Study Reveals Candidate Genes for Growth Relevant Traits in Pigs. Front. Genet..

[9] Cassady J.P., Young L.D., Leymaster K.A. (2002). Heterosis and recombination effects on pig growth and carcass traits. J. Anim. Sci..

[10] Visscher P., Pong-Wong R., Whittemore C., Haley C. (2000). Impact of biotechnology on (cross)breeding programmes in pigs. Livest. Prod. Sci..

[11] Fu L., Jiang Y., Wang C.L., Mei M.R., Zhou Z.W., Jiang Y.F., Song H.L., Ding X.D. (2020). A Genome-Wide Association Study on Feed Efficiency Related Traits in Landrace Pigs. Front. Genet..

[12] Wu P.X., Wang K., Yang Q., Zhou J., Liu D.J., Liu Y.H., Ma J.D., Tang Q., Jin L., Xiao W.H. (2019). Whole-genome re-sequencing association study for direct genetic effects and social genetic effects of six growth traits in Large White pigs. Sci. Rep..

[13] Jiang Y., Tang S., Wang C., Wang Y., Qin Y., Wang Y., Zhang J., Song H., Mi S., Yu F. (2018). A genome-wide association study of growth and fatness traits in two pig populations with different genetic backgrounds. J. Anim. Sci..

[14] Cantor R.M., Lange K., Sinsheimer J.S. (2010). Prioritizing GWAS Results. A Review of Statistical Methods and Recommendations for Their Application. Am. J. Hum. Genet..

[15] Korte A., Farlow A. (2013). The advantages and limitations of trait analysis with GWAS: A review. Plant Methods.

[16] Xue Y.H., Liu S., Li W.N., Mao R.H., Zhuo Y., Xing W.K., Liu J., Wang C., Zhou L., Lei M.G. (2022). Genome-Wide Association Study Reveals Additive and Non-Additive Effects on Growth Traits in Duroc Pigs. Genes.

[17] Li X., Zhang Y.B., Tang Z.L., Yu M., Liu B., Fan B., Xu S.P., Peng Z.Z., Zhao S.H., Zhu M.J. (2009). Comparative Estimation on Three-Way Heterosis in Pigs Reveals Genetic Bias of the Widely Used Empirical Formula. J. Anim. Vet. Adv..

[18] Toro M.A., Varona L. (2010). A note on mate allocation for dominance handling in genomic selection. Genet. Sel. Evol..

[19] Vitezica Z.G., Varona L., Legarra A. (2013). On the Additive and Dominant Variance and Covariance of Individuals Within the Genomic Selection Scope. Genetics.

[20] Su G.S., Christensen O.F., Ostersen T., Henryon M., Lund M.S. (2012). Estimating Additive and Non-Additive Genetic Variances and Predicting Genetic Merits Using Genome-Wide Dense Single Nucleotide Polymorphism Markers. PLoS ONE.

[21] Lopes M.S., Bastiaansen J.W.M., Harlizius B., Knol E.F., Bovenhuis H. (2014). A Genome-Wide Association Study Reveals Dominance Effects on Number of Teats in Pigs. PLoS ONE.

[22] Garcia A.A.F., Wang S.C., Melchinger A.E., Zeng Z.B. (2008). Quantitative Trait Loci Mapping and The Genetic Basis of Heterosis in Maize and Rice. Genetics.

[23] Liu Y., Long H., Feng S.M., Ma T.T., Wang M.F., Niu L.Z., Zhang X.Y., Wang L.N., Lei Y., Chen Y.L. (2021). Trait correlated expression combined with eQTL and ASE analyses identified novel candidate genes affecting intramuscular fat. BMC Genom..

[24] Terada K., Ohtani T., Ogawa S., Hirooka H. (2024). Genetic parameters for carcass and meat quality traits in Jinhua, Duroc, and their crossbred pigs. J. Anim. Breed Genet..

[25] Choi J.S., Jin S.K., Choi Y.I., Lee J.J. (2015). Effects of Duroc Breeding Lines on Carcass Composition and Meat Quality. Korean J. Food Sci. Anim. Resour..

[26] Wu Z.S., Wang Z.G., Wang P., Cheng L.Y., Li J.H., Luo Y.F., Yang L.F., Li L.F., Zeng J.H., Hu B. (2024). Integrative analysis of proteomics and lipidomic profiles reveal the fat deposition and meat quality in Duroc x Guangdong small spotted pig. Front. Vet. Sci..

[27] Purcell S., Neale B., Todd-Brown K., Thomas L., Ferreira M.A.R., Bender D., Maller J., Sklar P., de Bakker P.I.W., Daly M.J. (2007). PLINK: A tool set for whole-genome association and population-based linkage analyses. Am. J. Hum. Genet..

[28] Browning B.L., Tian X.W., Zhou Y., Browning S.R. (2021). Fast two-stage phasing of large-scale sequence data. Am. J. Hum. Genet..

[29] (2021). Maize Hoeci: Mid-Parent, Better-Parent and Standard Heterosis of Experimental Crosses in Maize. Int. J. Trop. Agric..

[30] Pérez P., de los Campos G. (2014). Genome-Wide Regression and Prediction with the BGLR Statistical Package. Genetics.

[31] Pérez-Rodríguez P., de los Campos G. (2022). Multitrait Bayesian shrinkage and variable selection models with the BGLR-R package. Genetics.

[32] VanRaden P.M. (2008). Efficient Methods to Compute Genomic Predictions. J. Dairy Sci..

[33] Hayes B.J., Visscher P.M., Goddard M.E. (2009). Increased accuracy of artificial selection by using the realized relationship matrix. Genet. Res..

[34] Zhou G.L., Xu F.J., Qiao J.K., Che Z.X., Xiang T., Liu X.L., Li X.Y., Zhao S.H., Zhu M.J. (2023). E-GWAS: An ensemble-like GWAS strategy that provides effective control over false positive rates without decreasing true positives. Genet. Sel. Evol..

[35] Yu J.M., Pressoir G., Briggs W.H., Bi I.V., Yamasaki M., Doebley J.F., McMullen M.D., Gaut B.S., Nielsen D.M., Holland J.B. (2006). A unified mixed-model method for association mapping that accounts for multiple levels of relatedness. Nat. Genet..

[36] Wang D., Tang H., Liu J.F., Xu S.Z., Zhang Q., Ning C. (2020). Rapid epistatic mixed-model association studies by controlling multiple polygenic effects. Bioinformatics.

[37] Segura V., Vilhjálmsson B.J., Platt A., Korte A., Seren Ü., Long Q., Nordborg M. (2012). An efficient multi-locus mixed-model approach for genome-wide association studies in structured populations. Nat. Genet..

[38] Liu X.L., Huang M., Fan B., Buckler E.S., Zhang Z.W. (2016). Iterative Usage of Fixed and Random Effect Models for Powerful and Efficient Genome-Wide Association Studies. PLoS Genet..

[39] Yin L.L., Zhang H.H., Tang Z.S., Xu J.Y., Yin D., Zhang Z.W., Yuan X.H., Zhu M.J., Zhao S.H., Li X.Y. (2021). rMVP: A Memory-efficient, Visualization-enhanced, and Parallel-accelerated Tool for Genome-wide Association Study. Genom. Proteom. Bioinf..

[40] Huang M., Liu X.L., Zhou Y., Summers R.M., Zhang Z.W. (2019). BLINK: A package for the next level of genome-wide association studies with both individuals and markers in the millions. Gigascience.

[41] Shaffer J.P. (1995). Multiple Hypothesis-Testing. Annu. Rev. Psychol..

[42] Cunningham F., Allen J.E., Allen J., Alvarez-Jarreta J., Amode M.R., Armean I.M., Austine-Orimoloye O., Azov A.G., Barnes I., Bennett R. (2022). Ensembl 2022. Nucleic Acids. Res..

[43] Mao X.Z., Cai T., Olyarchuk J.G., Wei L.P. (2005). Automated genome annotation and pathway identification using the KEGG Orthology (KO) as a controlled vocabulary. Bioinformatics.

[44] Wu J.M., Mao X.Z., Cai T., Luo J.C., Wei L.P. (2006). KOBAS server: A web-based platform for automated annotation and pathway identification. Nucleic Acids. Res..

[45] Wang J.B., Zhang Z.W. (2021). GAPIT Version 3: Boosting Power and Accuracy for Genomic Association and Prediction. Genom. Proteom. Bioinf..

[46] Muhammad A., Li J.G., Hu W.C., Yu J.S., Khan S.U., Khan M.H.U., Xie G.S., Wang J.B., Wang L.Q. (2021). Uncovering genomic regions controlling plant architectural traits in hexaploid wheat using different GWAS models. Sci. Rep..

[47] Liu S., Zhong H., Meng X.X., Sun T., Li Y.S., Pinson S.R.M., Chang S.K.C., Peng Z.H. (2020). Genome-wide association studies of ionomic and agronomic traits in USDA mini core collection of rice and comparative analyses of different mapping methods. BMC Plant Biol..

[48] Nida H., Girma G., Mekonen M., Tirfessa A., Seyoum A., Bejiga T., Birhanu C., Dessalegn K., Senbetay T., Ayana G. (2021). Genome-wide association analysis reveals seed protein loci as determinants of variations in grain mold resistance in sorghum. Theor. Appl. Genet..

[49] Ning C., Kang H.M., Zhou L., Wang D., Wang H.F., Wang A.G., Fu J.L., Zhang S.L., Liu J.F. (2017). Performance Gains in Genome-Wide Association Studies for Longitudinal Traits via Modeling Time-varied effects. Sci. Rep..

[50] Sahana G., Guldbrandtsen B., Thomsen B., Holm L.E., Panitz F., Brondum R.F., Bendixen C., Lund M.S. (2014). Genome-wide association study using high-density single nucleotide polymorphism arrays and whole-genome sequences for clinical mastitis traits in dairy cattle. J. Dairy Sci..

[51] Fowler K.E., Pong-Wong R., Bauer J., Clemente E.J., Reitter C.P., Affara N.A., Waite S., Walling G.A., Griffin D.K. (2013). Genome wide analysis reveals single nucleotide polymorphisms associated with fatness and putative novel copy number variants in three pig breeds. BMC Genom..

[52] Long Y., Ruan G.R., Su Y., Xiao S.J., Zhang Z.Y., Ren J., Ding N.S., Huang L.S. (2014). Genome-wide association study identifies QTLs for EBV of Backfat Thickness and average daily gain in Duroc pigs. Russ. J. Genet..

[53] Rogers S., Macheda M.L., Docherty S.E., Carty M.D., Henderson M.A., Soeller W.C., Gibbs E.M., James D.E., Best J.D. (2002). Identification of a novel glucose transporter-like protein-GLUT-12. Am. J. Physiol.-Endoc. Metab..

[54] White M.A., Tsouko E., Lin C.C., Rajapakshe K., Spencer J.M., Wilkenfeld S.R., Vakili S.S., Pulliam T.L., Awad D., Nikolos F. (2018). GLUT12 promotes prostate cancer cell growth and is regulated by androgens and CaMKK2 signaling. Endocr.-Relat. Cancer.

[55] Grapes L., Rothschild M.F. (2006). Investigation of a QTL region for loin eye area and fatness on pig Chromosome 1. Mamm. Genome.

[56] MacNeill S.A. (2010). Structure and function of the GINS complex, a key component of the eukaryotic replisome. Biochem. J..

[57] Das S.K., Ma L., Sharma N.K. (2015). Adipose tissue gene expression and metabolic health of obese adults. Int. J. Obes..

[58] Sajan S.A., Ganesh J., Shinde D.N., Powis Z., Scarano M.I., Stone J., Winter S., Tang S. (2019). Biallelic disruption of is associated with a skeletal disorder characterised by rhizomelic shortening of extremities and dysmorphic features. J. Med. Genet..

[59] Kinoshita M., Era T., Jakt L.M., Nishikawa S.I. (2009). The novel protein kinase Vlk is essential for stromal function of mesenchymal cells. Development.

[60] Miao Y.X., Mei Q.N., Fu C.K., Liao M.X., Liu Y., Xu X.W., Li X.Y., Zhao S.H., Xiang T. (2021). Genome-wide association and transcriptome studies identify candidate genes and pathways for feed conversion ratio in pigs. BMC Genom..

[61] Ibáñez-Zamacona M.E., Poveda A., Rebato E. (2019). Contribution of obesity associated genetic variants to anthropometric somatotype components. Anthr. Anz Ber Uber Biol-Anthr. Lit.

[62] Jiang L., Wang J., Wang K., Wang H., Wu Q., Yang C., Yu Y., Ni P., Zhong Y., Song Z. (2021). RNF217 regulates iron homeostasis through its E3 ubiquitin ligase activity by modulating ferroportin degradation. Blood.

[63] Song Q.Y., Meng X.R., Hinney A., Song J.Y., Huang T., Ma J., Wang H.J. (2018). Waist-hip ratio related genetic loci are associated with risk of impaired fasting glucose in Chinese children: A case control study. Nutr. Metab..

[64] Niu G.L., Yang Y.L., Zhang Y.Y., Hua C.J., Wang Z.S., Tang Z.L., Li K. (2016). Identifying suitable reference genes for gene expression analysis in developing skeletal muscle in pigs. PeerJ.

