# Supplementary material for: Additive and Dominance Genome-Wide Association Studies Reveal the Genetic Basis of Heterosis Related to Growth Traits of Duhua Hybrid Pigs
Source: Animals (Basel). 2024 Jun 30;14(13):1944. doi: 10.3390/ani14131944 (PMC11240614; doi:10.3390/ani14131944)
Supplement: Supplementary file 1 [file animals-14-01944-s001.zip › animals-3065885-supplementary.pdf]

Table S1. Functional annotation of candidate genes.

Term	Database	ID	Gene	P-Value
limb morphogenesis	Gene Ontology	GO:0035108	PKDCC	0.0022
negative regulation of fibroblast apoptotic process	Gene Ontology	GO:2000270	API5	0.0022
glucocorticoid receptor binding	Gene Ontology	GO:0035259	GRIP1	0.0022
neurotransmitter receptor transport, endosome to postsynaptic membrane	Gene Ontology	GO:0098887	GRIP1	0.0026
signaling receptor complex adaptor activity	Gene Ontology	GO:0030159	GRIP1	0.0030
embryonic digestive tract development	Gene Ontology	GO:0048566	PKDCC	0.0041
glucose transmembrane transport	Gene Ontology	GO:1904659	SLC2A12	0.0045
fibroblast growth factor binding	Gene Ontology	GO:0017134	API5	0.0048
positive regulation of chondrocyte differentiation	Gene Ontology	GO:0032332	PKDCC	0.0048
spindle assembly	Gene Ontology	GO:0051225	MAP9	0.0093
lung alveolus development	Gene Ontology	GO:0048286	PKDCC	0.0093
positive regulation of bone mineralization	Gene Ontology	GO:0030501	PKDCC	0.0097
dendrite development	Gene Ontology	GO:0016358	GRIP1	0.0108
bone mineralization	Gene Ontology	GO:0030282	PKDCC	0.0108
non-membrane spanning protein tyrosine kinase activity	Gene Ontology	GO:0004715	PKDCC	0.0130
mitotic cytokinesis	Gene Ontology	GO:0000281	MAP9	0.0134
spliceosomal complex	Gene Ontology	GO:0005681	API5	0.0141
DNA replication	Gene Ontology	GO:0006260	GINS3	0.0145
roof of mouth development	Gene Ontology	GO:0060021	PKDCC	0.0193
skeletal system development	Gene Ontology	GO:0001501	PKDCC	0.0204
multicellular organism growth	Gene Ontology	GO:0035264	PKDCC	0.0237
Aminoacyl-tRNA biosynthesis	KEGG PATHWAY	ssc00970	WARS2	0.0248
peptidyl-tyrosine phosphorylation	Gene Ontology	GO:0018108	PKDCC	0.0288
mitochondrial matrix	Gene Ontology	GO:0005759	WARS2	0.0346
transmembrane transporter activity	Gene Ontology	GO:0022857	SLC2A12	0.0425

Table S2. Summary statistics of phenotypic means for Duhua hybrid pigs and small spotted pigs of six groups.

Trait	Group	F1	Sow
100 AGE (day)	DD-YHSPW-19-400054	103.23 ± 3.92	178.43 ± 10.59
	DD-YHSPW-19-400077	102.07 ± 4.57	175.62 ± 8.21
	DD-YHSPW-19-400079	103.36 ± 3.76	196.65 ± 17.55
	DD-YHSPW-19-400071	105.10 ± 3.92	204.00
	DD-YHSPW-19-400078	101.74 ± 3.43	184.93 ± 11.93
	DD-YHSPW-19-400059	101.64 ± 3.53	162.55 ± 4.22
100 BF (mm)	DD-YHSPW-19-400054	26.32 ± 3.63	41.32 ± 4.42
	DD-YHSPW-19-400077	24.78 ± 4.20	43.29 ± 2.27
	DD-YHSPW-19-400079	22.54 ± 3.72	37.23 ± 1.22
	DD-YHSPW-19-400071	19.64 ± 1.64	26.28
	DD-YHSPW-19-400078	23.00 ± 3.12	40.43 ± 4.89
	DD-YHSPW-19-400059	24.35 ± 3.30	35.57 ± 2.57

Notes: Group, six groups (divided by paternity); F1, phenotypic statistics (mean ± standard deviation) of Duhua hybrid pigs; Sow, phenotypic statistics (mean ± standard deviation) of small spotted pigs.
